# Compressed Sensing Diffusion Spectrum Imaging for Accelerated Diffusion Microstructure MRI in Long-Term Population Imaging

**DOI:** 10.3389/fnins.2018.00650

**Published:** 2018-09-24

**Authors:** Alexandra Tobisch, Rüdiger Stirnberg, Robbert L. Harms, Thomas Schultz, Alard Roebroeck, Monique M. B. Breteler, Tony Stöcker

**Affiliations:** ^1^German Center for Neurodegenerative Diseases, Bonn, Germany; ^2^Department of Computer Science, University of Bonn, Bonn, Germany; ^3^Department of Cognitive Neuroscience, Faculty of Psychology and Neuroscience, Maastricht University, Maastricht, Netherlands; ^4^Bonn-Aachen International Center for Information Technology, University of Bonn, Bonn, Germany; ^5^Faculty of Medicine, Institute for Medical Biometry, Informatics and Epidemiology, University of Bonn, Bonn, Germany; ^6^Department of Physics and Astronomy, University of Bonn, Bonn, Germany

**Keywords:** diffusion MRI, diffusion spectrum imaging, compressed sensing, multi-shell HARDI, microstructure, population imaging

## Abstract

Mapping non-invasively the complex microstructural architecture of the living human brain, diffusion magnetic resonance imaging (dMRI) is one of the core imaging modalities in current population studies. For the application in longitudinal population imaging, the dMRI protocol should deliver reliable data with maximum potential for future analysis. With the recent introduction of novel MRI hardware, advanced dMRI acquisition strategies can be applied within reasonable scan time. In this work we conducted a pilot study based on the requirements for high resolution dMRI in a long-term and high throughput population study. The key question was: can diffusion spectrum imaging accelerated by compressed sensing theory (CS-DSI) be used as an advanced imaging protocol for microstructure dMRI in a long-term population imaging study? As a minimum requirement we expected a high level of agreement of several diffusion metrics derived from both CS-DSI and a 3-shell high angular resolution diffusion imaging (HARDI) acquisition, an established imaging strategy used in other population studies. A wide spectrum of state-of-the-art diffusion processing and analysis techniques was applied to the pilot study data including quantitative diffusion and microstructural parameter mapping, fiber orientation estimation and white matter fiber tracking. When considering diffusion weighted images up to the same maximum diffusion weighting for both protocols, group analysis across 20 subjects indicates that CS-DSI performs comparable to 3-shell HARDI in the estimation of diffusion and microstructural parameters. Further, both protocols provide similar results in the estimation of fiber orientations and for local fiber tracking. CS-DSI provides high radial resolution while maintaining high angular resolution and it is well-suited for analysis strategies that require high *b*-value acquisitions, such as CHARMED modeling and biomarkers from the diffusion propagator.

## 1. Introduction

Diffusion magnetic resonance imaging (dMRI) provides, completely non-invasively, unique insights into the complex microstructural architecture of the living human brain. Sensitized for the random motion of water molecules, dMRI offers a distinct imaging contrast to investigate the diffusion process at a microscopic scale (Le Bihan et al., 1986). A whole range of mathematical representations (e.g., Basser et al., 1994; Jensen et al., 2005; Wedeen et al., 2005; Ozarslan et al., 2009 and biophysical models, e.g., Behrens et al., 2003; Assaf and Basser, 2005; Zhang et al., 2012), exists to characterize the diffusion signal as well as the underlying microstructure and to infer macroscopic brain connections from voxel-wise fiber orientation estimates. This makes dMRI a powerful imaging modality to study *in vivo* pathological changes of diffusion in brain tissue and the influence of disease on the structural connectivity of brain white matter (WM).

The increasing life expectancy in modern society and its consequences for the public health sector give rise to a growing number of population studies that have been set up in the last decades [e.g., Rotterdam study, (Breteler et al., 1994; De Groot et al., 2000), Alzheimer's Disease Neuroimaging Initiative (ADNI), (Weiner et al., 2015), Human Connectome Project (HCP), (Glasser et al., 2016), UK Biobank study, (Miller et al., 2016), Rhineland Study, (Stöcker, 2016)] to acquire rich data from their participants with the aim to provide new insights into disease development and progression and to discover biomarkers for disease prediction at an early state and potentially its prevention, but also for health promotion in general. As a potential biomarker for brain diseases dMRI is, thus, a natural fit for being one of the core imaging protocols in population imaging. Long-term population studies require the dMRI protocol to deliver reliable data with maximum potential for future analysis. Therefore, an extensive pilot phase is the starting point of such studies to define the acquisition strategies based on suggestions from state-of-the-art research. For dMRI, decisions with respect to the diffusion protocol must be made on e.g., spatial resolution, q-space sampling, acceleration strategies, phase encoding directions and many more. DMRI analysis techniques further influence the choice of the imaging protocol by imposing special requirements on the dMRI acquisitions for accurate data processing.

Since the 1990s population studies have been investigating brain changes due to disease by means of neuroimaging aiming for large sample sizes and using standard MR scanners with the purpose of acquiring imaging data comparable to that of other clinical studies (Ikram et al., 2015; Weiner et al., 2015; Miller et al., 2016). Due to common MR hardware and scan time limitations, diffusion tensor imaging (Basser et al., 1994) (DTI) or 2-shell HARDI is performed in these studies for the collection of dMRI acquisitions. With the aim to provide very high quality diffusion data, the two consortia of the Human Connectome (Behrens and Sporns, 2012; Jbabdi et al., 2015) Project use highly customized MR scanners to improve diffusion imaging by very strong magnetic field gradients with a maximum amplitude of 100 mT/m (Van Essen et al., 2012; Sotiropoulos et al., 2013) or even 300 mT/m (Setsompop et al., 2013). In this context, advanced dMRI protocols such as multi-shell high angular resolution diffusion imaging (Tuch et al., 2002) (HARDI), high *b*-value q-Ball imaging (Tuch, 2004; Wu and Alexander, 2007) and diffusion spectrum imaging (Wedeen et al., 2005) (DSI) are employed to collect high resolution dMRI acquisitions in about 1 h of acquisition time (McNab et al., 2013; Sotiropoulos et al., 2013; Fan et al., 2016). The bespoke systems developed in this context have paved the way for a new generation of clinical MRI systems with higher maximum gradient strength up to 80 mT/m (Glasser et al., 2016). With these scanners' introduction the advantages of the aforementioned studies can now be combined, meeting both the aim for high quality diffusion data as well as the scan time limitation in high throughput population studies. Thus, MRI scanners with a powerful gradient system allow for high resolution diffusion imaging by means of advanced dMRI protocols within a reasonable scan time. Following the well-established HCP dMRI protocol (Sotiropoulos et al., 2013), a 3-shell HARDI protocol is a natural candidate for being the dMRI protocol of choice in this setting. However, advances in the development of novel acquisition strategies for fast collection of dMRI scans that provide high resolution of intra-voxel microstructure indicate that diffusion spectrum imaging (Wedeen et al., 2005) (DSI) accelerated by the application of the compressed sensing (Menzel et al., 2011; Bilgic et al., 2012; Setsompop et al., 2013; Paquette et al., 2015; Tobisch et al., 2015) (CS) theory also has high potential to fit the task of time efficient versatile diffusion imaging.

In this work we conducted a pilot study specifically designed based on the requirements for a long-term population study, the Rhineland Study, to investigate the performance of CS-DSI for the acquisition of high resolution dMRI data at 3T. The CS-DSI protocol was time-matched with a state-of-the-art 3-shell HARDI protocol that runs 12 minutes in total. As part of the 1-h MR protocol in the Rhineland Study (Stöcker, 2016), the dMRI scheme should deliver data with maximum potential for future analysis to enable both microstructure imaging and fiber tracking and thereby tractometry (Bells et al., 2011; De Santis et al., 2014b), as well as connectomics analysis. Thus, a wide spectrum of state-of-the-art diffusion processing and analysis techniques was applied to the pilot study acquisitions. The results of this work validate the applicability of DSI accelerated with compressed sensing for population imaging and highlight the potentials of this imaging protocol in the context of a long-term population study.

## 2. Materials and methods

### 2.1. DMRI acquisition

#### 2.1.1. Diffusion MR imaging protocols

**Figure 1:**
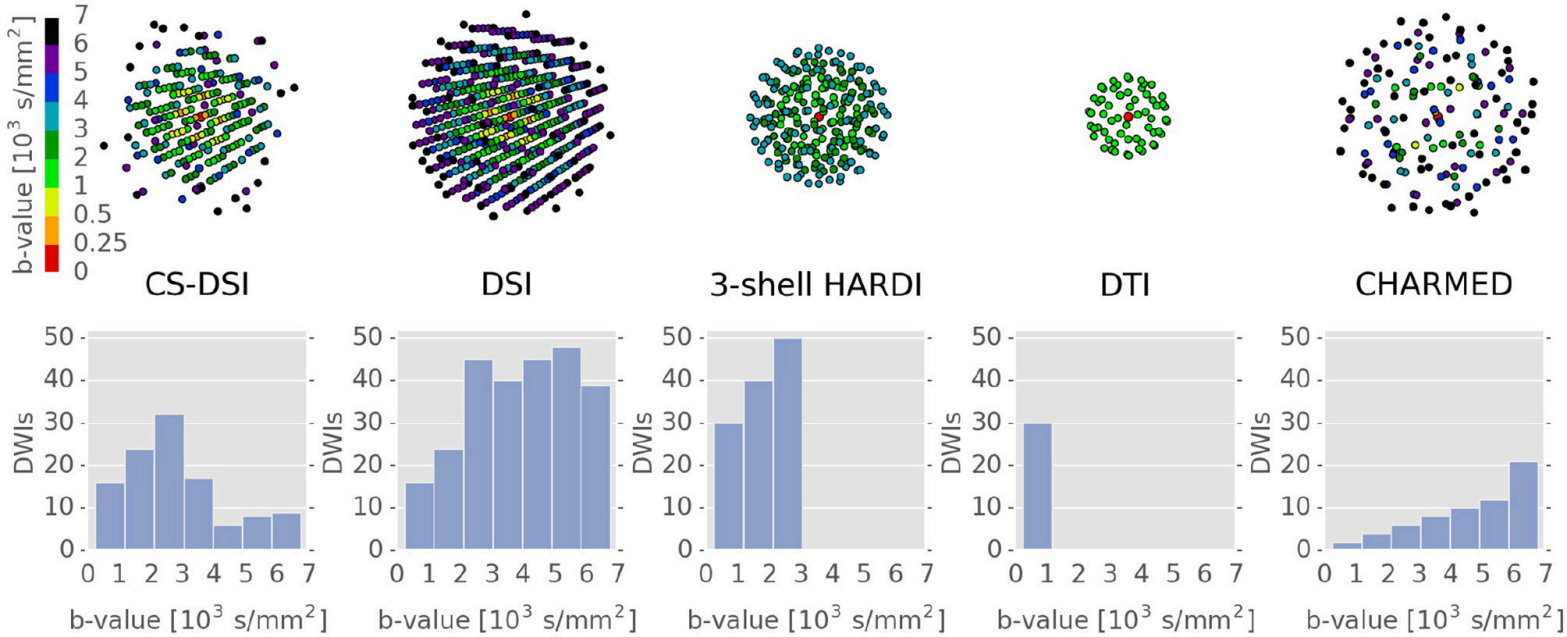
DMRI sampling distributions and diffusion weighting of the advanced imaging protocols CS-DSI and 3-shell HARDI and of DTI and CHARMED schemes. **(Top row)** The color coding of the q-space samples highlights the range of *b*-values specific to each protocol. The number of depicted q-space samples per protocol corresponds to the sum of acquired DWIs and their antipodal counterparts. **(Bottom row)** The number of DWIs acquired per protocol is depicted as a function of the *b*-value. In contrast to the single (DTI) and the multi-shell (3-shell HARDI) scheme, CS-DSI, DSI and CHARMED contain high *b*-value acquisitions.

Based on previous works (Sotiropoulos et al., 2013; Paquette et al., 2015; Tobisch et al., 2015), we adapted and optimized a CS-DSI and a 3-shell HARDI protocol for dMRI at 3T. Figure 1 depicts the three-dimensional q-space sampling and the corresponding *b*-value distribution of both advanced protocols in comparison to dedicated sampling schemes for DTI and CHARMED (Assaf and Basser, 2005). 3-shell HARDI samples the q-space with high angular resolution and provides adequate radial resolution by incorporating three different diffusion-weightings at *b*-values of 1,000, 2,000, and 3,000 s/mm^2^. In addition to 14 interleaved *b* = 0 scans, these three q-space shells respectively contain 30, 40, and 50 samples that are optimally distributed for an advantageous uniform angular q-space coverage (De Santis et al., 2014a; Sprenger et al., 2016) following the design proposed by Caruyer et al. (2013). In total, 120 diffusion weighted images (DWIs) are acquired using 3-shell HARDI. Our choice of a HARDI scheme with three shells at *b*-values of 1,000, 2,000, and 3,000 s/mm^2^ is based on recent literature on comparable variants of multi-shell schemes. Several works report the suitability of those schemes for population imaging (Sotiropoulos et al., 2013) and their advantages for optimal diffusion parameter extraction by means of microstructure models (Alexander and Barker, 2005; Poot et al., 2010; Kamath et al., 2012; Zhang et al., 2012; Sprenger et al., 2016) and for the estimation of orientational information (Kamath et al., 2012; Sotiropoulos et al., 2013; Tournier et al., 2013). In our work, we, therefore, consider 3-shell HARDI as a gold standard protocol for multi-shell imaging in population studies to which we aim to compare CS-DSI. In contrast to the multi-shell protocol, DSI requires a uniform Cartesian grid sampling scheme, typically of size 11 × 11 × 11 truncated to a sphere, which leads to a total number of 258 unique samples in q-space covering the latter at both high angular and high radial resolution. One drawback of traditional DSI is the long acquisition time. However, recent advances in combining DSI with CS theory allow for accelerated imaging by reducing the MR acquisition to fewer q-space samples sufficient for subsequent recovery of the full data by means of non-linear reconstruction. Exploiting the antipodal symmetry of the diffusion signal and applying an acceleration factor of 2.3, the CS-DSI protocol, thus, acquires 112 DWIs with diffusion weightings in the range of *b* = 270–6,800 s/mm^2^ plus 8 interleaved *b* = 0 scans. The b-vectors and *b*-values applied for CS-DSI and 3-shell HARDI data acquisition are provided as Supplementary Material. We select the maximum *b*-value of the CS-DSI scheme in accordance with recent literature on conventional and CS-accelerated DSI (Bilgic et al., 2012; Gigandet et al., 2013; Paquette et al., 2015; Yeh and Verstynen, 2016) and recommendations for microstructure imaging requiring high *b*-values such as CHARMED modeling (De Santis et al., 2014a). The CS-DSI acquisition scheme was generated based on the design proposed by Paquette et al. (2015). All samples follow a uniform angular distribution and cover q-space randomly in the radial direction (Jones et al., 1999; Paquette et al., 2015) subject to Cartesian discretization within the 11 × 11 × 11 truncated sphere. Based on simulation experiments, no significant difference in CS reconstruction was observed across 100 randomly generated instances of this sampling scheme for 2.3-fold CS acceleration (Tobisch et al., 2015). By means of CS reconstruction of the undersampled DSI acquisitions, all 515 samples on the q-space grid are recovered forming the basis for the following DSI data analysis. As state-of-the-art reference protocols, we further consider a dedicated CHARMED protocol containing 72 q-space samples in the range of *b* = 850–6,800 s/mm^2^ (De Santis et al., 2014a) as well as a DTI scheme with 30 DWIs at *b*-value 1,000 s/mm^2^. The latter is represented by the inner shell of the 3-shell HARDI sampling scheme similarly to other works that utilize a suitable subset of the acquired multi-shell data for fitting the tensor model (Sotiropoulos et al., 2013; Miller et al., 2016).

The selection of a suitable q-space sampling scheme only covers one aspect in the process of designing a diffusion protocol. The following section will summarize the key decisions made during a first sequence development stage with respect to the dMRI acquisition in the context of scan time-limited population imaging. All dMRI scans are collected on a 3T Siemens MAGNETOM Prisma MRI scanner (Siemens Healthcare, Erlangen, Germany) equipped with a powerful gradient system with maximum amplitudes of 80 mT/m and slew rates of 200 mT/m/ms as well as a 64-channel head-neck coil. We perform accelerated diffusion imaging by means of a simultaneous-multi-slice (SMS) dMRI sequence employing threefold slice-acceleration (Setsompop et al., 2012; Xu et al., 2013; Cauley et al., 2014). No in-plane acceleration is applied (GRAPPA R = 1) in combination with multiband excitation. Facilitated by the performant gradient system, a relatively large readout bandwidth of 1,785 Hz/pixel is selected to counteract increased geometric distortions along the phase encode direction at 1.5 mm resolution. All imaging protocols apply monopolar diffusion weighting and a partial Fourier factor of 6/8 to minimize TE. All sequence parameters are matched across protocols with identical spatial resolution, except for the gradient pulse separation (Δ), the gradient pulse duration (δ), the echo time (TE) and the repetition time (TR). Allowing for small differences, the latter parameters are optimized separately for each protocol to match the time requirement of the population study while still providing comparable diffusion contrast among the imaging protocols of interest. Slightly higher values for TE and TR due to higher *b*-value acquisitions result in a reduced number of CS-DSI scans and a slightly reduced signal-to-noise ratio (SNR) in comparison to the 3-shell HARDI protocol. For all dMRI acquisitions, TR and TE are kept constant across q-space samples to avoid effects through noise levels varying with the diffusion weighting and T2 relaxation, which would prevent data processing with state-of-the-art diffusion analysis tools. Running straight-forward data analysis outweighs the increase in SNR achievable by optimizing TE and TR separately for different *b*-values. Due to powerful MRI hardware and advanced diffusion sequences acquiring dMRI scans at a higher resolution than 2.0 mm isotropic is feasible and likewise preferred for diffusion imaging in renowned population studies (Sotiropoulos et al., 2013).

#### 2.1.2. Diffusion pilot study

Diffusion MRI scans were acquired from 20 healthy subjects (age range 20–77, mean age 47.5, 5 males, 15 females) at 1.5 mm isotropic resolution using the two advanced imaging protocols: CS-DSI (TE/TR = 101.4 ms/5,300 ms, Δ = 49.5 ms, δ = 19.7 ms) and 3-shell HARDI (TE/TR = 90 ms/4,800 ms, Δ = 43.9 ms, δ = 14 ms). In addition, reference scans were acquired with a dedicated 1.5mm isotropic CHARMED protocol (TE/TR = 101.4 ms/5,300 ms, Δ = 49.5 ms, δ = 19.7 ms) in 4 of the subjects. Approval to undertake the study was obtained from the ethics committee of the University of Bonn, Medical Faculty. The study was carried out in accordance with the recommendations of the International Council for Harmonization (ICH) Good Clinical Practice (GCP) standards (ICH-GCP). We obtained written informed consent from all participants in accordance with the Declaration of Helsinki. The scan time per subject was 11 min for the 3-shell and the CS-DSI protocol, supporting the applicability in population imaging. One minute of additional *b* = 0 scans, including an autocalibration scan, with reversed phase encoding (PE) polarity were collected per protocol. Further, rescans are collected on the same day, but only for one specific imaging protocol per subject. In total, 5 rescans were acquired for each imaging protocol of interest. In addition to dMRI, a rapid, 1 mm isotropic T1-weighted MP-RAGE scan (Brenner et al., 2014) was acquired per subject within 3 minutes at the end of the first scanning session (TI = 1,100 ms, TE/TR = 2.83 ms/2,530 ms). For dMRI analysis, the acquisitions of all 20 subjects are divided into two distinct subsets: the scans of 16 subjects for the investigation of CS-DSI and 3-shell HARDI (Group A) and the scans of the remaining 4 subjects for the comparison of these protocol to the CHARMED reference protocol (Group B).

### 2.2. DMRI preprocessing

#### 2.2.1. Motion and distortion correction

All images are corrected for subject motion and distortions prior to the estimation of diffusion and microstructural parameters. FSL's topup is used to estimate susceptibility-induced geometric distortions (Andersson et al., 2003) whereas FSL's eddy is applied to simultaneously correct for those distortions as well as eddy-current-induced artifacts and subject motion (Andersson and Sotiropoulos, 2015). However, eddy is not designed for DSI data as it requires the dMRI samples to be acquired on shells in q-space. For a fair comparison of both protocols of interest, independently of differences between processing algorithms, we aim to use the same motion and distortion correction. Thus, we apply a *post hoc* adjustment of the nominal DSI *b*-values according to the requirements of eddy such that the DSI q-space samples acquired on a Cartesian grid shift by maximal 50 s/mm^2^ to *b*-values corresponding to samples on 20 shells in q-space. This enables the Gaussian process modeling in eddy. Note that the adjustments only affect the nominal *b*-values not the actual *b*-values nor the CS-DSI acquisitions. After motion and distortion correction, the original, unshifted *b*-values are used for all subsequent processing steps. To validate this approach, we additionally acquired dMRI data from one healthy subject using the 3-shell HARDI and the CS-DSI imaging protocol. We have extended the 3-shell HARDI scheme by 6 uniformly distributed DWIs at *b* = 700 s/mm^2^ to allow for an adequate comparison to corresponding CS-DSI DWIs with b < 1,000 s/mm^2^. Instead of just collecting a few *b* = 0 images with reversed phase encoding polarity as in the pilot study, the complete protocols are run twice, once with anterior-to-posterior (AP) and once with posterior-to-anterior (PA) PE direction. In this way, eddy becomes applicable for DSI data without the nominal *b*-value shifts. To assess the performance of eddy, the mean squared error (MSE) between corresponding images of the AP and PA data sets is calculated before and after eddy correction and for CS-DSI, additionally with and without the shift of nominal *b*-values. In the pilot study, DWIs are only acquired with AP PE direction and thus motion and distortion correction is performed with the help of a few *b* = 0 scans with reversed phase encoding polarity. To simulate a scenario similar to the pilot study, the quality of those corrected AP PE DWIs is evaluated by calculating the MSE with respect to the PA PE DWIs that were corrected using both AP and PA PE data.

#### 2.2.2. Compressed sensing in dMRI

Compressed sensing (CS) reconstruction was applied to recover the diffusion propagator **p** from the undersampled DSI acquisitions **y** by means of the discrete Fourier transform *ℱ* combined with a sparsity term (Paquette et al., 2015; Tobisch et al., 2015):

(1) $$\begin{array}{l} \underset{\text{p}\in R^{n}}{\mathrm{argmin}}||{\text{R}}_{\Omega }ℱ\text{p}-\text{y}|{|}_{l_{2}}+\lambda ||\text{p}|{|}_{l_{1}} \end{array}$$

The CS matrix **R**_**Ω**_*ℱ* incorporates the undersampling matrix **R**_**Ω**_. λ is the empirically determined regularization parameter set to 5 × 10^−6^. The *l*_2_-norm and *l*_1_-norm are applied to the data consistency and the sparsity term, respectively. In this setting, the diffusion propagator itself defines a natural sparsity domain well-suited for CS reconstruction (Merlet and Deriche, 2010). For the complete DSI data recovery, an iterative shrinkage and thresholding algorithm (Gong et al., 2013) solves the convex optimization problem. Here, we do not validate or describe in more detail the CS theory as this exceeds the scope of the paper. For the interested reader, we refer to the corresponding literature on applications of CS in dMRI, specifically CS-DSI (Menzel et al., 2011; Bilgic et al., 2012; Setsompop et al., 2013; Tobisch et al., 2015; Paquette et al., 2016). We choose the degree of CS acceleration to be relatively small to ensure good CS reconstruction quality and thus, selected a modest acceleration factor of 2.3. CS reconstruction generates 257 unique DWIs from the 112 measurements fully describing the diffusion propagator in DSI. For brevity, we will refer to this set of 257 reconstructed DWIs as the CS-DSI data from now on. We also considered sparse reconstruction for the multi-shell scheme. Several recent works on CS for spherical sampling strategies exist (Michailovich et al., 2011; Rathi et al., 2014; Fick et al., 2016). Having run an extensive evaluation study of CS methods for sparse reconstruction, Ning et al. (2015) report that signal reconstruction with the MAPL MRI model proposed by Fick et al. (2016) allows signal extrapolation at higher *b*-values and performs similar to the CS approach by Rathi et al. (2014). We considered the former CS method which is implemented as part of the Dipy library (Garyfallidis et al., 2014). The 3-shell HARDI, CS-DSI, and CHARMED data of Group B were used to reconstruct the respective signals for the diffusion directions of the CHARMED reference protocol with *b*-values up *b* = 6,800 s/mm^2^. To compare the reconstruction quality, we computed the normalized mean square error (NMSE) as defined in Ning et al. (2015) with respect to the CHARMED reference signal of both 3-shell HARDI and CS-DSI. For the analysis of CS-DSI and 3-shell HARDI acquisitions in the context of population imaging, we decided against sparse reconstruction for the multi-shell scheme, the reasons for which will be discussed later on. Thus, in this work, all dMRI analysis is performed on 3-shell HARDI and CS-DSI acquisitions with CS reconstruction only done for the latter.

### 2.3. DMRI analysis

#### 2.3.1. Diffusion and microstructural parameter estimation

Using the Maastricht Diffusion Toolbox (Harms et al., 2017) and in-house implementations, several mathematical and biophysical diffusion models were fitted to the data to estimate the following diffusion and microstructure parameters: fractional anisotropy (FA) and mean, axial and radial diffusivity (MD, AD, RD) from the tensor model (Basser et al., 1994), mean, axial and radial kurtosis (MK, AK, RK) as well as FA, MD, AD, and RD from the kurtosis model (Jensen et al., 2005), the weight (volume fraction) of the intra-cellular compartment (wIC) and orientation dispersion (ODI) from the NODDI model (Zhang et al., 2012), and intra-axonal restricted volume fraction (FR) from the CHARMED model (Assaf and Basser, 2005). Each diffusion model comes with specific requirements or recommendations for the maximum or optimal *b*-values of the dMRI data. Considering these specifications, we fit the tensor, kurtosis and NODDI model to DWIs acquired with *b*-values of approx. 1,000 s/mm^2^, up to 3,000 s/mm^2^ and on two shells of approx. 1,000 and 3,000 s/mm^2^, respectively. The CHARMED model specifically requires acquisitions of *b*-values greater than 3,000 s/mm^2^. Table 1 lists the selection and the resulting total number of unique DWIs based on the *b*-value requirements of specific diffusion models and highlights the differences in the model fitting process between 3-shell HARDI and CS-DSI acquisitions. For CS-DSI, additional maps of mean squared displacement (MSD), return-to-origin-probability (RTOP) (Wu et al., 2008) and non-Gaussianity (NG) (Özarslan et al., 2013) are derived from the diffusion propagator by means of the MAPL MRI model.

**Table 1:** Selection and resulting total number of unique DWIs for the CS-DSI and the 3-shell HARDI protocol based on the *b*-value requirements of the tensor, kurtosis, NODDI and CHARMED model.

		**3-shell HARDI**	**CS-DSI**
DT	b [s/mm^2^]	1,000	270–1,300
	DWIs	30	28
Kurtosis	b [s/mm^2^]	1,000–3,000	270–3,000
	DWIs	120	85
NODDI	b [s/mm^2^]	1,000, 3,000	270–1,300, 3,000–3,800
	DWIs	30, 50	28, 52
CHARMED	b [s/mm^2^]	n/a	270–6,800
	DWIs	n/a	257

As the basis for statistical group analysis, the standard tract-based spatial statistics (TBSS) routine was applied to project all estimated diffusion parameters to a mean FA skeleton (Smith et al., 2006). The TBSS framework is well established and likewise preferred in other works (Zhu et al., 2015; Giezendanner et al., 2016) also in combination with atlas-based identification of WM tracts (Santis et al., 2012). We acknowledge, however, the reported limitations of TBSS (Edden and Jones, 2011; Bach et al., 2014). Thus, for population-based analysis, alternative analysis techniques should be investigated. Regions of interest (ROIs) were automatically defined by mapping the labels of well-known white matter tracts in the brain provided in standard space by the JHU ICBM DTI 81 atlas (Mori et al., 2008) available in FSL to the TBSS mean FA skeleton. We selected nine ROIs of well-known WM tracts for statistical analysis: the splenium, body and genu of the corpus callosum (SCC, BCC, GCC), the anterior limb of internal capsule (ALIC), the sagittal stratum (SS), the superior corona radiata (SCR), the corticospinal tracts (CST), the superior longitudinal fasciculus (SLF) and the superior fronto-occipital fasciculus (SFO). Left (L) and right (R) regions are considered for all tracts, except the CC. For each diffusion parameter, the group mean and standard error (SEM) were subsequently calculated from the mean across all voxels within the tract ROIs. To provide further statistical information, we performed voxel-wise analysis of paired two-group differences using FSL's randomize (5,000 permutations, TFCE, variance smoothing) (Winkler et al., 2014). Additional statistical analysis was performed to investigate effects across WM structures. For each diffusion parameter, we used a linear fixed effects model with random intercept for each model in order to account for the clustering of the measurements within each subject. As fixed effects we included acquisition scheme and WM ROI. This statistical analysis was performed using R Statistical Software (version 3.4.1) and the lme4 package.

To quantify the test-retest (TRT) reliability of a diffusion imaging protocol, we assessed the differences between diffusion parameters estimated from the scans and rescans of a subgroup of subjects from the pilot study. Per subject, diffusion parameter maps of both scan and rescan were, first, linearly registered to a mid-space to account for the influence of resampling effects on the data that could mask TRT variability in the case of registering one scan to the other. Second, the difference between the registered parameter maps as well as their mean was calculated and warped into MNI space. Finally, the parameter specific mean and differences for each subject were projected onto the WM skeleton and subsequent ROI based analysis was performed as for the group analysis described before. To asses the reliability and repeatability of the 3-shell HARDI and the CS-DSI diffusion protocol, the intraclass correlation coefficient (ICC) and the within-subject coefficient of variation (wsCV) were calculated, respectively (Bartko, 1966; Bland and Altman, 1996).

#### 2.3.2. Fiber orientation and macroscopic brain connections

Fiber orientation distribution functions (fODFs) were obtained by means of the recently proposed method by Ankele et al. (2017) which incorporates the SHORE (Ozarslan et al., 2009) model that continuously describes the diffusion signal. The novel SHORE-based multi tissue constrained spherical deconvolution (CSD) approach by Ankele et al. (2017), here denoted as the SHORE/MT-CSD model, can be applied independently of the dMRI sampling scheme. It allows the generation of brain tissue volume fraction maps for white matter, gray matter (GM) and cerebrospinal fluid (CSF) and the estimation of fODFs for both CS-DSI and 3-shell HARDI. fODF estimation as proposed by Ankele et al. (2017) requires T1 imaging data for tissue segmentation similar to Jeurissen et al. (2014). In contrast, state-of-the-art methods for optimal fiber orientation estimation that depend on specific q-space sampling exist for both CS-DSI and 3-shell HARDI: for CS-DSI, diffusion ODFs (dODFs) can be directly calculated from the DSI diffusion propagator (Wedeen et al., 2005; Paquette et al., 2016); the high angular resolution of HARDI data is most advantageous for estimating fiber orientation by means of CSD providing the fiber ODF instead of the diffusion ODF (Tournier et al., 2007). All ODFs were reconstructed using either the approach by Ankele et al. (2017) or the Dipy library (Garyfallidis et al., 2014) and visualized using MATLAB. For a voxel-wise qualitative as well as quantitative comparison of the orientational information provided by both advanced dMRI protocols, CS-DSI and 3-shell HARDI acquisitions are registered to a mid-space prior to ODF estimation. To avoid any influence of methodological differences in the ODF estimation on the quantitative analysis, we consider the fODFs estimated via (Ankele et al., 2017) for a quantitative comparison of CS-DSI and 3-shell HARDI. Ankele et al. (2017) recommend a SHORE order of 4 for fODF estimation. For an optimal visualization, we additionally compute the fODFs with SHORE order 8. Complementing the visual fODF comparison, we performed voxel-wise analysis for fODFs with SHORE order 8 by means of the angular cross-correlation (Anderson, 2005). Further analysis was conducted on fODFs with SHORE order 4. We compare the CS-DSI and the 3-shell HARDI scheme with respect to the angular difference between corresponding fODF peak directions as well as the deviation between crossing angles of the two most dominant fiber directions in voxels with at least two fibers. To restrict the analysis mainly to pure WM voxels, we followed the recommendations by Jeurissen et al. (2013). The minimum crossing angle that occurs in at least 1% of the WM voxels was determined for each scheme. We also compute the percentages of voxels containing one, two or three dominant fiber fascicles (1/2/3 fiber voxels) within the applied WM mask. For this purpose, we define the number of detected fibers per voxel as the minimum number of fibers that are detected by both schemes with a corresponding partial WM volume fraction greater than 0.1. To extend the investigation of 3-shell HARDI and CS-DSI from voxel-wise ODFs to macroscopic brain connections, we used FSL's bedpostx and probtrackx in combination with the autoPtx plugin (Behrens et al., 2007; De Groot et al., 2013). Those tools provide a standard pipeline for probabilistic tractography to generate path probability maps for distinct WM tracts in the brain. For both 3-shell HARDI and CS-DSI, tract-specific probability maps were averaged across all subjects and thresholded for visualization based on the recommendations in De Groot et al. (2015).

## 3. Results

### 3.1. DMRI preprocessing

#### 3.1.1. Motion and distortion correction

**Figure 2:**
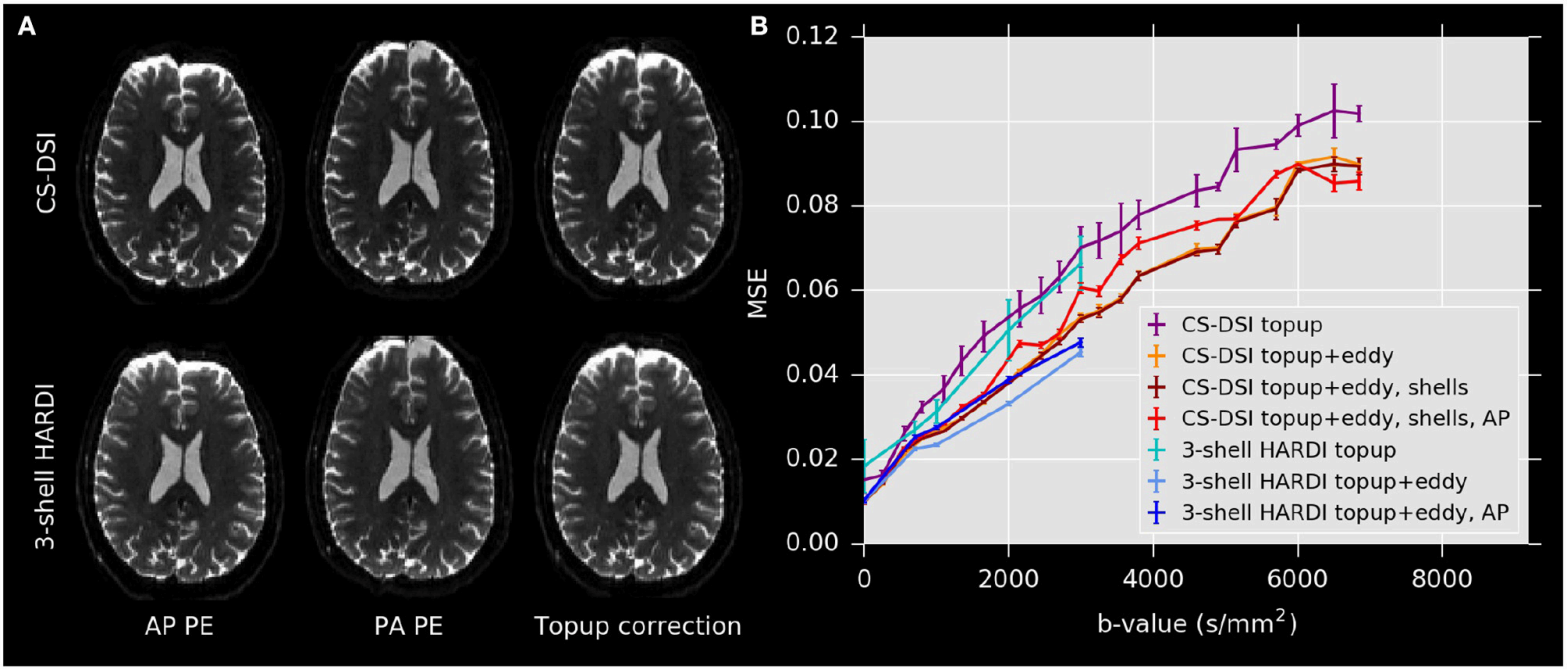
**(A)** Example *b* = 0 images with opposite PE directions and following topup correction for CS-DSI and 3-shell HARDI. **(B)** MSE calculated for the simulated pilot study scenario (“AP”) and between corresponding images acquired with both AP and PA PE directions before and after eddy correction as well as with (“shells”) and without a nominal *b*-value shift for CS-DSI acquisitions.

Figure 2A demonstrates that for both CS-DSI and 3-shell HARDI, susceptibility-induced distortions are corrected successfully using FSL's topup. Eddy-current-induced artifacts as well as subject motion were corrected using FSL's eddy. The applicability of this approach for DSI acquisitions as introduced above is validated by calculating the MSE between corresponding images acquired with AP and PA PE directions before and after eddy correction. Figure 2B depicts comparable MSE for dMRI data with and without the small *post hoc* shift of nominal *b*-values (“shells”). Further, for the calculated deviations in AP and PA PE measurements as well as the simulated pilot study scenario, which utilizes only AP PE DWIs (“AP”), both diffusion protocols, overall, yield similar differences between the MSE before and after eddy correction. However, Figure 2B shows that in the lower *b*-value regime the reduction in MSE due to eddy correction is higher for CS-DSI than for 3-shell HARDI, whereas in the higher *b*-value regime the opposite trend is observed. Specifically, considering the simulated pilot study scenario, where only AP sampling of *b* > 0 is used: up to *b* = 2,000 s/mm^2^ the MSE curves of both CS-DSI and 3-shell HARDI largely overlap, and for instance at *b*-values of approx. 1,000 s/mm^2^, the MSE reduction is 25 and 12% for CS-DSI and 3-shell HARDI, respectively. However, at *b* = 3,000 s/mm^2^ considerably higher MSE values are observed for CS-DSI. Accordingly, the MSE reduction is only 14% for CS-DSI compared to 28% for 3-shell HARDI. The same holds for eddy correction based on AP and PA PE measurements. At low *b*-values, the MSE reduction is 31% for CS-DSI and 25% for 3-shell HARDI. For higher *b*-values the MSE is reduced by 24 and 32%, respectively. The AP and PA PE eddy-current correction approach leads to smallest MSE values for 3-shell HARDI for all *b*-values up to 3,000 s/mm^2^.

#### 3.1.2. Compressed sensing in dMRI

**Figure 3:**
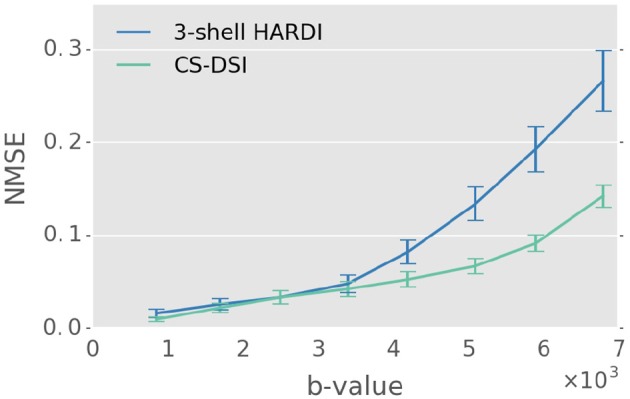
For 3-shell HARDI, CS-DSI, and CHARMED data of Group B the respective signals for the diffusion directions of the CHARMED reference protocol were reconstructed using the MAPL MRI model. The NMSE with respect to the CHARMED reference signal was computed as a function of the *b*-value for both 3-shell HARDI and CS-DSI. Superior reconstruction quality is observed for CS-DSI in the *b*-value regime above 4,000 s/mm^2^.

Figure 3 depicts the NMSE with respect to the CHARMED reference signal of both 3-shell HARDI and CS-DSI. In general, the reconstruction error increases with increasing *b*-value. In the *b*-value regime below 4,000 s/mm^2^, a low NMSE of about 5% is observed for both 3-shell HARDI and CS-DSI. However, for 3-shell HARDI, signal extrapolation at higher *b*-values leads to an NMSE of up to 30% and is, thus, inferior compared to the signal interpolation for CS-DSI.

### 3.2. DMRI analysis

#### 3.2.1. Diffusion and microstructural parameter estimation

**Figure 4:**
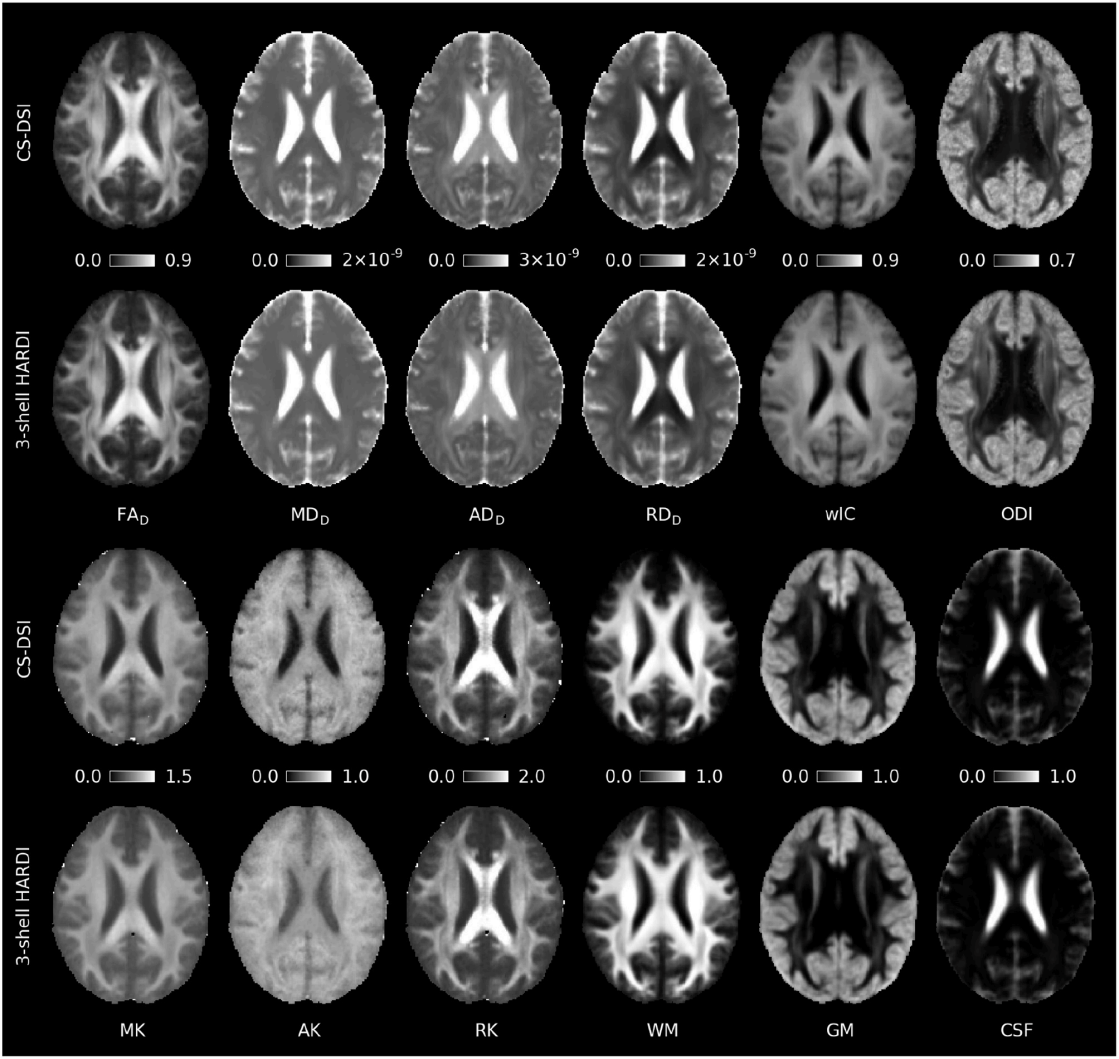
Group averaged whole brain diffusion parameter maps estimated from CS-DSI and 3-shell HARDI acquisitions by means of the tensor (FA_D_, MD_D_, AD_D_, RD_D_), kurtosis (MK, AK, RK), and NODDI (wIC, ODI) model. Brain tissue volume fraction maps for WM, GM, and CSF were estimated using multi-tissue deconvolution. Comparable parameter maps are obtained for both CS-DSI and 3-shell HARDI. Diffusivities are given in units of m^2^/s.

Group averaged whole brain diffusion parameter maps obtained by means of the tensor (FA_D_, MD_D_, AD_D_, RD_D_), kurtosis (MK, AK, RK), and NODDI (wIC, ODI) model are visualized in Figure 4. We denote FA and diffusivities derived from the tensor model by the subscript D, those from the kurtosis model by the subscript K. Figure 4 further presents tissue volume fraction maps for WM, GM, and CSF obtained for both CS-DSI and 3-shell HARDI using the approach by Ankele et al. (2017). To generate the parameter maps, MR scans from the subset of 16 subjects (Group A) were used. SNR is slightly lower for CS-DSI due to higher *b*-value acquisitions. However, no significant difference between the group averaged parameter maps is noticeable by visual comparison of CS-DSI and 3-shell HARDI.

**Figure 5:**
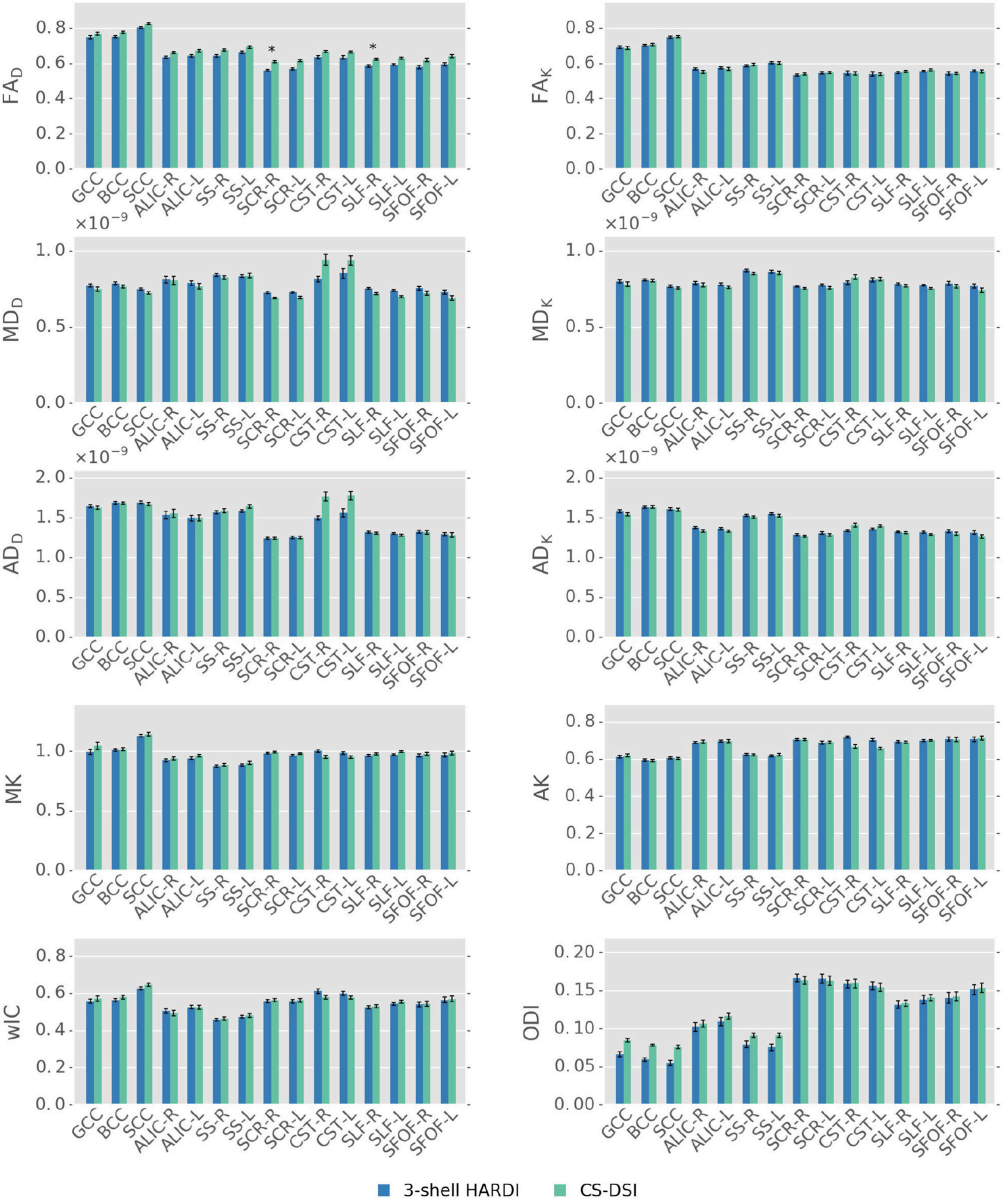
Parameter specific group mean and standard error for the tensor (FA_D_, MD_D_, AD_D_), kurtosis (FA_K_, MD_K_, AD_K_, MK, AK), and NODDI (wIC, ODI) model. For each WM ROI, mean *p*-values were extracted from the respective skeleton and are, if significant (*p* < 0.025), indicated as: *p < 0.025 and **p < 0.005. Except for FA derived from the tensor model, the analysis of significant differences in diffusion parameters estimated from CS-DSI and 3-shell HARDI acquisitions indicates comparable performance of both schemes. Diffusivities are given in units of m^2^/s.

**Figure 6:**
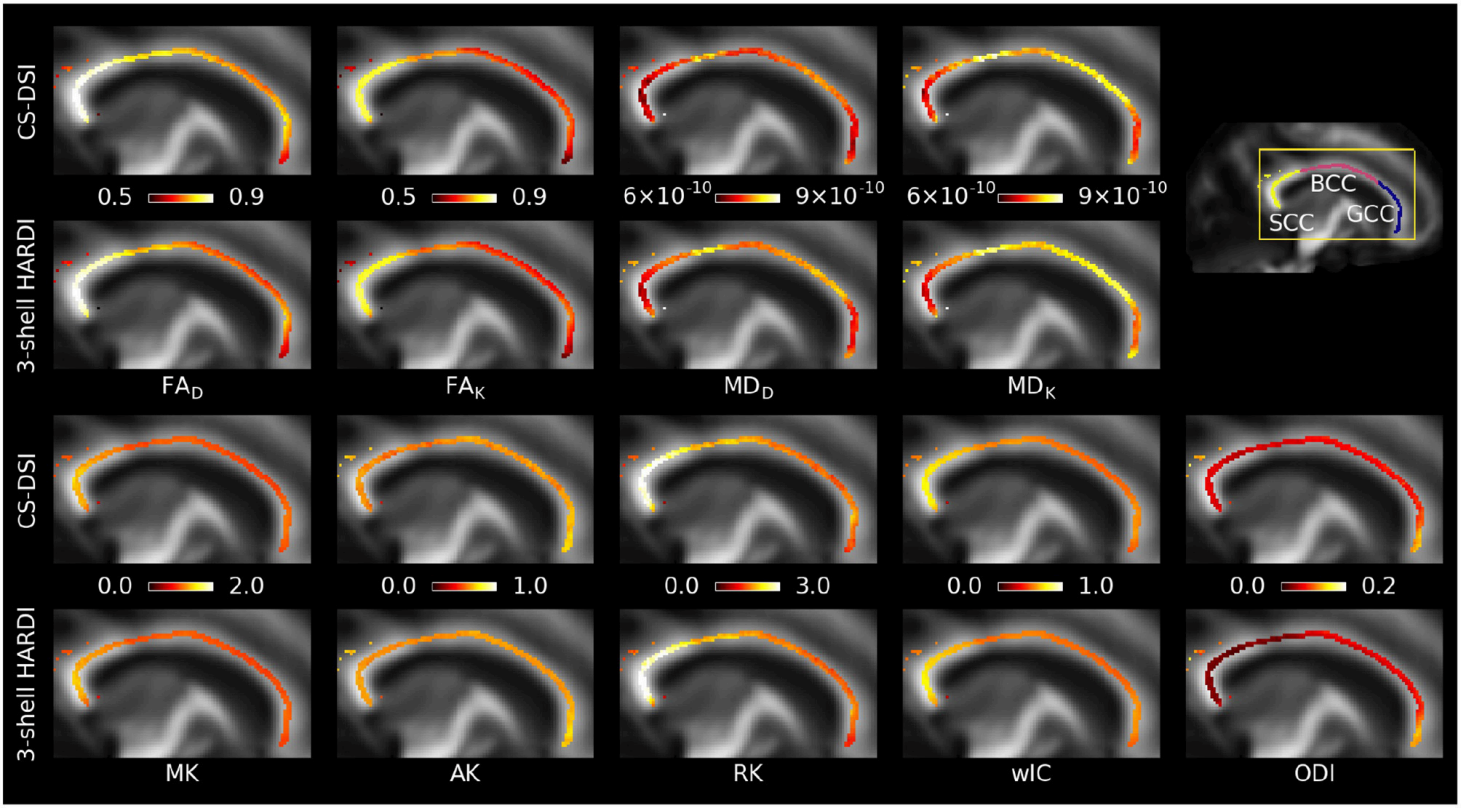
Sagittal view of group mean values across CC voxels on the TBSS white matter skeleton for parameters estimated from the tensor (FA_D_, MD_D_), kurtosis (FA_K_, MD_K_, MK, AK, RK), and NODDI (wIC, ODI) model overlaid on the FA map in WM atlas space. **(Top two rows)** Kurtosis model fitting reduces the differences in FA and MD between CS-DSI and 3-shell HARDI compared to the tensor model. **(Bottom two rows)** Comparable kurtosis and NODDI metrics are derived for both acquisition schemes. Diffusivities are given in units of m^2^/s.

For parameters derived from the tensor (FA_D_, MD_D_, AD_D_), kurtosis (FA_K_, MD_K_, AD_K_, MK, AK, RK), and NODDI (wIC) model, the group mean and standard error calculated across all voxels on the TBSS white matter skeleton within each of the nine WM tract ROIs are shown in Figure 5. For each WM ROI, Figure 5, further, indicates the statistical significance (*p* < 0.025) of the differences between CS-DSI and 3-shell HARDI in the estimated diffusion parameters obtained from FSL's randomize. To this end, the mean *p*-value was extracted from the respective skeleton within each WM ROI (Vovk and Wang, 2012). A linear fixed effects model was run for each diffusion parameter depicted in Figure 5 and the resulting *p*-value and the effect size with confidence intervals are reported in Table 2. The significance level of these 1-tailed tests was Bonferroni corrected for multiple comparisons and set to 0.025/N, where *N* = 10 is the number of the independent linear fixed effects models. Significant differences in CS-DSI and 3-shell HARDI are observed for FA using both FSL's randomize and the fixed effects model. For the latter, significant differences are also observed in AD_D_ and ODI. To further explore differences in estimated diffusion parameters between the two imaging protocols, we determined residual errors of the MDT model fitting by computing the NMSE for the tensor and the NODDI model fitted to CS-DSI and 3-shell HARDI acquisitions. For the tensor model, the average NMSE across voxels within the WM ROIs was 0.027 ± 0.013 and 0.022 ± 0.010 for CS-DSI and 3-shell HARDI, respectively. For the NODDI model, the average NMSE was 0.060 ± 0.016 and 0.053 ± 0.015 for CS-DSI and 3-shell HARDI, respectively. Figure 6 provides more details on the analysis of the three subregions of the CC by depicting the group mean across CC voxels on the skeleton calculated for parameters estimated from the tensor (FA_D_, MD_D_), kurtosis (FA_K_, MD_K_, MK, AK, RK) and NODDI (wIC, ODI) model. The results presented in Figures 5, 6 indicate similar performance of both the CS-DSI and the 3-shell HARDI imaging protocol in the estimation of FA, MD and AD from both the tensor and the kurtosis model as well as MK, AK, RK, wIC, and ODI. For the tensor model, a slight upward FA bias is noticeable for CS-DSI compared to 3-shell HARDI, which is reduced through the application of the kurtosis model. Differences in mean diffusivity between the protocols are less significant for MD_K_ in comparison with MD_D_. Comparable kurtosis and NODDI metrics are derived for both acquisition schemes, except for ODI estimated in the CC regions. Overall, differences in mean and standard error between CS-DSI and 3-shell HARDI are smaller than differences in the diffusion parameter values between different WM tracts.

**Table 2:** The effect size and the corresponding confidence interval obtained from a linear mixed effects model for each of the diffusion parameter depicted in Figure 5 is provided together with the degree of significance.

FA_*D*_	3.38 × 10^−2^	(2.95 × 10^−2^ to 3.80 × 10^−2^)^****^
MD_*D*_	−7.94 × 10^−12^	(−1.80 × 10^−11^ to 2.11 × 10^−12^)
AD_*D*_	3.21 × 10^−11^	(1.38 × 10^−11^ to 5.05 × 10^−11^)^**^
FA_*K*_	2.66 × 10^−4^	(−3.97 × 10^−3^ to 4.50 × 10^−3^)
MD_*K*_	−1.12 × 10^−11^	(−1.66 × 10^−11^ to −5.79 × 10^−12^)
AD_*K*_	−1.51 × 10^−11^	(−2.41 × 10^−11^ to −6.15 × 10^−12^)
MK	9.08 × 10^−3^	(1.96 × 10^−3^ to 1.62 × 10^−2^)
AK	−5.67 × 10^−3^	(−9.95 × 10^−3^ to −1.39 × 10^−3^)
wIC	2.08 × 10^−3^	(−2.97 × 10^−3^ to 7.13 × 10^−3^)
ODI	6.59 × 10^−3^	(3.87 × 10^−3^ to 9.31 × 10^−3^)^****^

The significance level of these 1-tailed tests was Bonferroni corrected for multiple comparisons and set to 0.025/N, where N is the number of the independent linear fixed effects models. If significant (p < 0.025/N), p-values are indicated as: ^*^p < 0.025/N,

** *p < 0.005/N*,

^***^p < 0.0005/N and

**** *p < 0.00005/N*.

**Figure 7:**
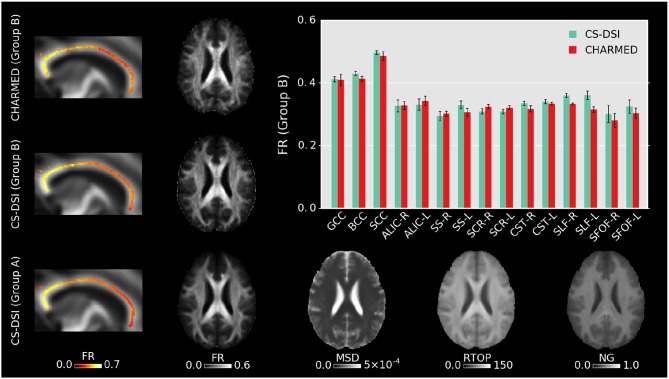
Group averaged whole brain FR maps, FR across the CC skeleton and the FR group mean and standard error across the skeleton within well-known WM ROIs are obtained by fitting the CHARMED model to CS-DSI (Group A and B) and CHARMED (Group B) acquisitions. Both CS-DSI and CHARMED schemes incorporate high *b*-value acquisitions that allow for accurate CHARMED modeling. For CS-DSI, additional maps of MSD, RTOP, and NG are obtained from the diffusion propagator by means of the MAPL MRI model. MSD and RTOP are given in units of mm^2^ and mm^−1^, respectively.

FR was estimated by fitting the CHARMED model to CS-DSI (Group A and B) and dedicated CHARMED (Group B) acquisitions. Figure 7 compares the resulting group averaged FR maps and shows additional maps of MSD, RTOP and NG derived from the diffusion propagator by means for the MAPL MRI model. Here, RTOP is visualized as the return-to-origin-probability to the power of 1/3 (Özarslan et al., 2013). Similar to Figures 5 and 6, Figure 7 further depicts the group mean and standard error of the group-specific FR calculated across all voxels on the skeleton within each WM ROI and provides a detailed visualization for the three subregions of the CC. Overall, FR obtained from CS-DSI is close to the CHARMED reference.

**Figure 8:**
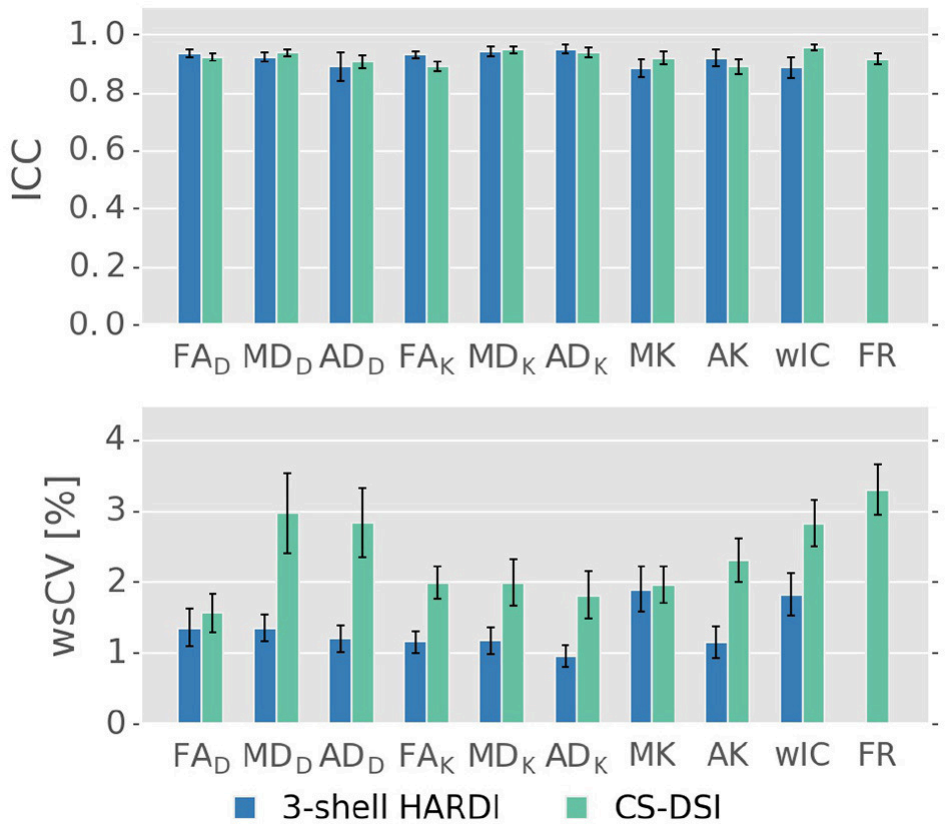
Parameter specific ICC and wsCV averaged over nine WM tracts for the tensor (FA_D_, MD_D_, AD_D_), kurtosis (FA_K_, MD_K_, AD_K_, MK, AK), NODDI (wIC), and CHARMED (FR) model. The latter is only reported for CS-DSI. ICC values present similar reliability for both imaging protocols. For CS-DSI, slightly lower repeatability is indicated by higher wsCV compared to 3-shell HARDI.

The test-retest reliability and repeatability of both diffusion imaging protocols of interest is validated by means of ICC and wsCV, respectively. Figure 8 depicts the TRT results for FA, MD and AD from both the tensor and the kurtosis model as well as MK, AK, wIC and FR as the average across the nine WM tracts and its standard error. For both protocols, diffusion parameter specific ICC and wsCV values greater than 0.85 and less than 4%, respectively, lie within acceptable ranges (Vollmar et al., 2010; Willats et al., 2014). The ICC values present similar reliability for both imaging protocols. For 3-shell HARDI acquisitions, wsCV increases for diffusion metrics of more advanced diffusion models. For CS-DSI, slightly lower repeatability is indicated by higher wsCV compared to 3-shell HARDI, however, repeatability of diffusivities improves with the use of the kurtosis model.

#### 3.2.2. Fiber orientation and macroscopic brain connections

**Figure 9:**
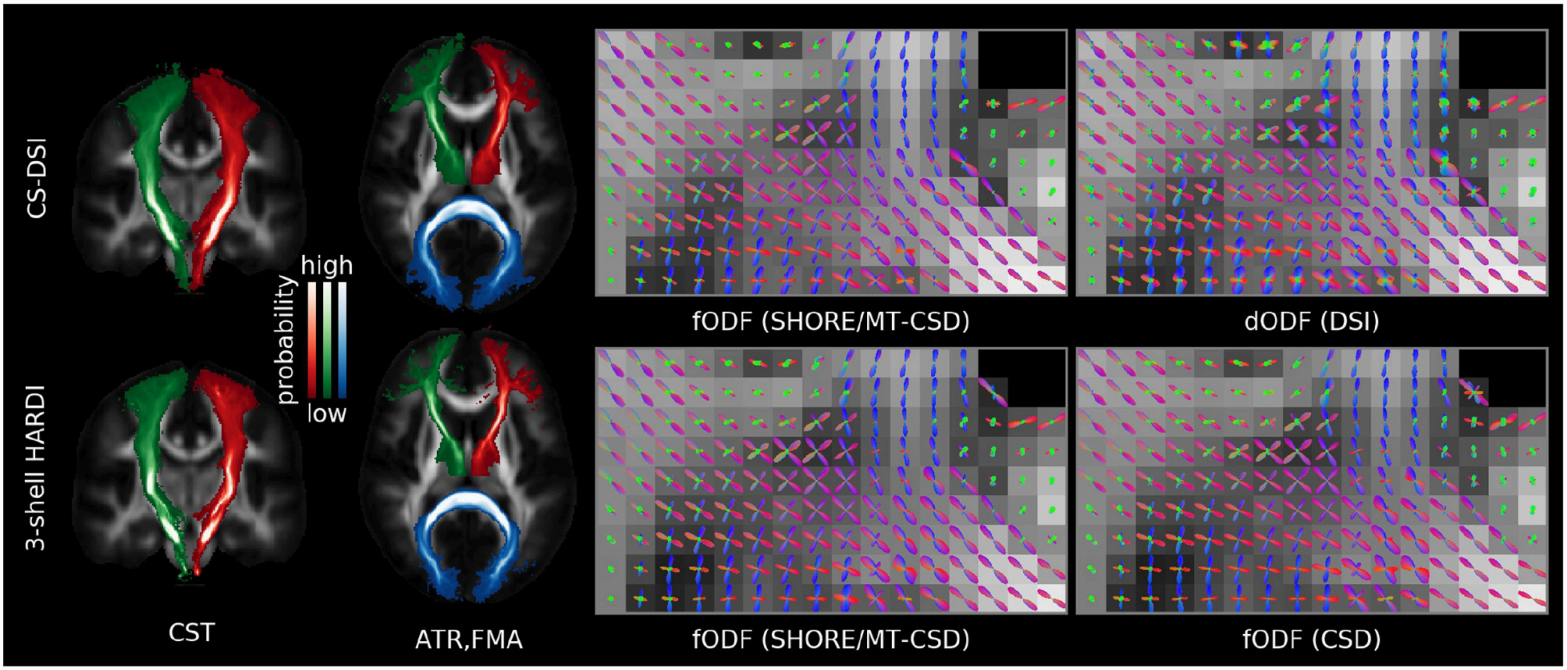
Comparable orientational information can be extracted from both CS-DSI and 3-shell HARDI acquisitions. **(Left)** Group averaged tract probability maps are superimposed on the JHU ICBM FA white matter atlas for the left (red) and right (green) corticospinal tract (CST), the left (red) and right (green) anterior thalamic radiation (ATR) and in blue the forceps major (FMA). **(Right)** Single-subject fODFs obtained by means of the SHORE/MT-CSD model (SHORE order 8) for both CS-DSI and 3-shell HARDI and, only for the latter, by means of the CSD model. Single-subject dODFs are derived from the DSI propagator.

**Figure 10:**
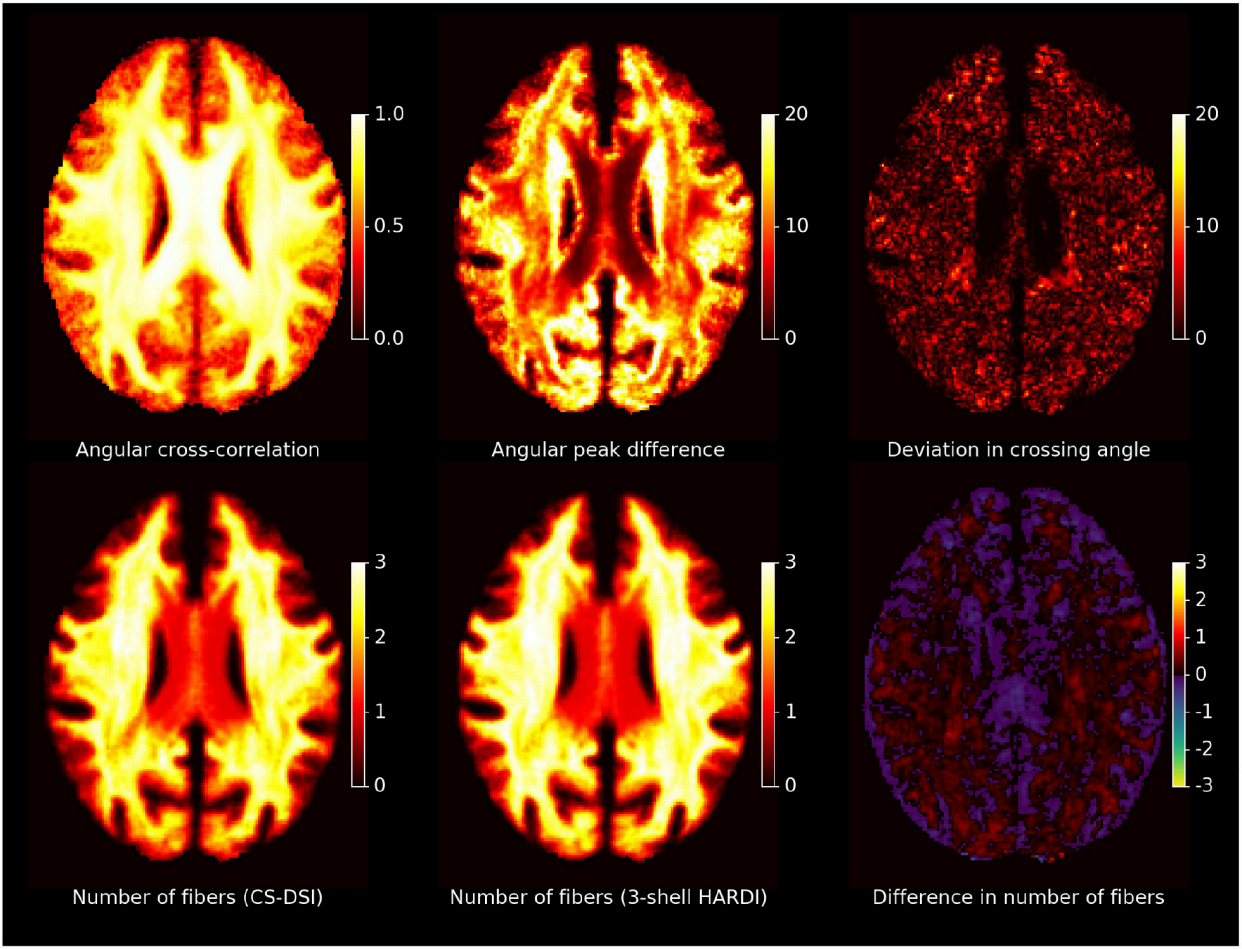
Spatial maps of the orientational information obtained from CS-DSI and 3-shell HARDI acquisitions: An axial slice of group-averaged whole brain maps of the angular cross correlation, the angular peak difference, the deviation in crossing angle and the number of fibers per imaging scheme and the corresponding difference (3-shell HARDI−CS-DSI) is shown. All metrics are computed for fODFs estimated with SHORE order 4, except for the angular cross correlation (SHORE order 8). The angular peak difference and the deviation in crossing angle are reported in degrees.

Figure 9 visualizes group averaged tract probability maps overlaid on the WM atlas. Representative of sensorimotor, association and commissural tracts, the left (red) and right (green) corticospinal tract (CST), the left (red) and right (green) anterior thalamic radiation (ATR) and in blue the forceps major (FMA) are selected, respectively. The tracking results for CS-DSI and 3-shell HARDI show similar visitation counts for streamlines of the CST, ATR and FMA. Note that for the CST no lateral projections are shown due to the pre-defined target mask of the autoPtx plugin. Additionally, Figure 9 depicts single-subject diffusion and fiber ODFs in a coronal ROI of crossing fibers from the CC, CST, and SLF. By visual comparison, diffusion ODFs calculated from the diffusion propagator indicate similar orientational information to that of fODFs obtained from CS-DSI and 3-shell HARDI. Comparable fODFs estimated via (Ankele et al., 2017) are obtained for both CS-DSI and 3-shell HARDI. This finding is supported by the quantitative analysis results presented in Table 3. Across all subjects, a high angular cross-correlation of 0.93 is observed with low standard error, as well as a small angular deviation of 9.7° and 7.8° in the estimated fiber directions and in the crossing angle in multi-fiber voxels, respectively. The minimum crossing angle that occurs in at least 1% of the WM voxels, is, on average, as low as 28.7° for CS-DSI and 29.1° for 3-shell HARDI. Percentages of 15.6/32.9/51.6 are determined for 1/2/3 fiber voxels within the applied WM mask. To support the quantitative analysis of the orientational information obtained from CS-DSI and 3-shell HARDI acquisitions (Table 3), Figure 10 provides maps of the group-averaged angular cross correlation, angular peak difference, deviation in crossing angle and number of fibers for an axial slice. For the latter, the number of fibers estimated in both CS-DSI and 3-shell HARDI and the difference of both maps (3-shell HARDI−CS-DSI) are shown.

**Table 3:** Quantitative analysis of the orientational information obtained from CS-DSI and 3-shell HARDI acquisitions: Group mean and standard error are reported for the angular cross correlation of both schemes, the angular deviation in the estimated fiber directions and in the crossing angle in multi-fiber voxels, the minimum crossing angle resolved by each scheme and the percentages of 1/2/3 fiber voxels within the WM mask.

	**Mean**	**SEM**
Angular cross-correlation	0.931	0.006
Angular peak difference	9.7	0.4
Deviation in crossing angle	7.8	0.3
Min. crossing angle (CS-DSI/3-shell)	28.7/29.1	0.2/0.4
Percentage of 1/2/3 fiber voxels	15.6/32.9/51.6	0.6/0.9/1.3

*All metrics are computed for fODFs estimated with SHORE order 4, except for the angular cross correlation (SHORE order 8)*.

## 4. Discussion

This work investigates CS-DSI for accelerated diffusion microstructure MRI in population imaging. For this purpose, we consider a 3-shell HARDI acquisition scheme as a gold standard protocol for multi-shell imaging in population studies, to which CS-DSI is compared to. We aimed to implement the comparison of these two conceptually very different diffusion protocols as even as possible, but we do not expect perfect agreement of diffusion parameters estimated from 3-shell HARDI and CS-DSI acquisitions due to differences in: the dMRI sequence parameters, even though the differences were kept minimal, subject motion during acquisitions and the selection of specific DWIs based on the *b*-value requirements for diffusion model fitting. Keeping these effects in mind, qualitative as well as quantitative dMRI analysis, overall, indicates comparable performance of CS-DSI and 3-shell HARDI on both single-subject and group level.

### 4.1. DMRI preprocessing

#### 4.1.1. Motion and distortion correction

Considering 3-shell HARDI data to be the reference for FSL's eddy, the validation results of our approach to apply FSL's eddy to DSI data, overall, indicate adequate and similar correction of susceptibility-induced and eddy-current-induced distortions and motion artifacts in CS-DSI acquisitions. However, our validation experiment still captured differences in eddy correction between CS-DSI and 3-shell HARDI. They suggest that, in the low *b*-value regime, eddy correction leads to more reduction in MSE for CS-DSI compared to 3-shell HARDI. At higher *b*-values, on the other hand, the reduction in MSE due to eddy correction is lower for CS-DSI than for 3-shell HARDI and the final MSE level is considerably lower for 3-shell HARDI at *b* = 3,000 s/mm^2^. The latter effect might be caused due to the sparse sampling distribution for CS-DSI at high *b*-values. Note, however, that this experiment was only performed with a single subject and for two different acquisitions for CS-DSI and 3-shell HARDI. Thus, the graphs presented in Figure 2 do not allow us to draw conclusions at a significant level. Within the scope of this work, using the same and preferably state-of-the-art processing tools for all data is a requirement for a reasonable comparison between the 3-shell HARDI and CS-DSI imaging protocol. Nevertheless, we notice that this approach might perform in favor of 3-shell HARDI acquisitions and improved corrections could be achieved with tools specifically tailored for CS-DSI acquisitions. This work's overall comparable results for 3-shell HARDI and CS-DSI, however, show no preference of this processing step toward multi-shell data or any negative effect on subsequent dMRI analysis. Note, that we have tried to support motion estimation across low and high *b*-values by interleaving *b* = 0 measurements every 14 and 10 DWIs for CS-DSI and 3-shell HARDI, respectively. Nevertheless, we acknowledge that motion estimation becomes increasingly difficult the higher the *b*-value gets. We currently investigate the use of integrated free induction decay navigators (FIDnav) (Kober et al., 2011) as an independent means to estimate rigid-body head motion parameters. Such a navigator signal can be acquired within less than 3 ms before each excitation and diffusion weighting (Kober et al., 2012). Ongoing improvements (Stirnberg et al., 2016; Wallace et al., 2018) may potentially make purely FIDnav-informed prospective motion correction possible.

#### 4.1.2. Compressed sensing in dMRI

In the context of population imaging, it has been shown before that CS-DSI provides comparable results to conventional DSI (Setsompop et al., 2012). A general concern with CS-DSI is the risk of missing subtle details about the diffusion process due to insufficient angular q-space sampling. However, the results of this work support that the risk of a dMRI protocol to not fully capture all diffusion information is not increased for CS-DSI accelerated by moderate undersampling because comparable diffusion parameters are estimated for both CS-DSI and 3-shell HARDI. Prior experiments based on full DSI simulations, phantom and *in vivo* measurements also confirmed the accurate CS reconstruction at such modest acceleration factors. For a single subject and 2.3-fold CS acceleration, for instance, the angular cross-correlation and peak difference both calculated between full and CS reconstructed DSI data over the brain WM are 0.964 and 5.3°, respectively. Fortunately, about 2-fold q-space undersampling already enables CS-DSI acquisitions within a similar time frame as 3-shell HARDI. Combining CS with an undersampling factor of 2.3 and SMS acquisition with an MB factor of 3 results in about 7-fold accelerated dMRI acquisitions compared to traditional DSI. CS-DSI scans, thus, can be acquired within the scan time limits of high throughput population studies.

In our work, we did consider sparse reconstruction for the 3-shell HARDI scheme, but decided against it for the following reasons. Ning et al. (2015) thoroughly investigated CS reconstruction methods as well as the degree of CS acceleration for multi-shell imaging. They recommend the CS approaches by Rathi et al. (2014) and Fick et al. (2016) for accurate signal reconstruction and suggest a CS factor similar to the one applied for CS-DSI in this work. Thus, our choice on the degree of undersampling for CS-DSI complies with other works in this research field. However, we do not apply the CS approach by Rathi et al. (2014) since (1) staggered q-space sampling is preferred over collinear acquisition schemes for diffusion and microstructure modeling (De Santis et al., 2014a; Sprenger et al., 2016) and (2) more thorough investigations are recommended to ensure incorporating CS reconstruction based on dictionaries is suitable for population imaging (Bilgic et al., 2012). And (3), realizing a target angular resolution corresponding to 120 directions per shell (the resolution of the staggered 3-shell scheme) with an undersampling factor of 2.3 would require a collinear sampling with 50 directions per shell. I.e., only two shells would be feasible in our scan time limit, or barely three shells, if we sacrificed target angular resolution (e.g., 92 = 2.3 × 40 directions per shell). Note, however, that this case would not allow for additional sparse radial reconstruction without exceeding a total undersampling factor of 2.3.

Subsampling the established HCP protocol containing 90 directions per shell was investigated using the CS approach by Fick et al. (2016). Applying a CS factor of 2.3 results in about 120 DWIs which is the number of diffusion weighted acquisitions we chose for the time-matched 3-shell HARDI protocol in this work. We use the approach by Fick et al. (2016) for signal interpolation of the 3-shell HARDI as well as CS-DSI data to obtain information of the diffusive transport along different gradient directions than those applied during image acquisition. As depicted in Figure 3, the reference signal can be adequately reconstructed for both 3-shell HARDI and CS-DSI as indicated by an NMSE of about 5% which is similar to the results reported by Ning et al. (2015). Thus, both the 3-shell HARDI as well as the CS-DSI data can be further enriched by reconstructing additional DWIs across the chosen *b*-value regime via the approach by Fick et al. (2016). Such investigations of increasing the angular as well as radial resolution are highly interesting but beyond the scope of this work.

A limitation of this work is the choice of different maximum *b*-values for CS-DSI and 3-shell HARDI. We selected the well-established 3-shell HARDI scheme as a gold standard protocol for multi-shell imaging in population studies, as investigating a novel design of the 3-shell HARDI scheme as outlined above exceeds the scope of this work. We decided against including shells at higher *b*-values for the 3-shell HARDI scheme since, to comply with the scan time limits of our study, this adaptation would come at the cost of a reduced number of samples at lower *b*-value shells, which would increase the CS factor, or the exclusion of shells, which we do not consider for the reasons discussed before. We aimed to address this limitation of our work by applying the CS approach by Fick et al. (2016) to extrapolate high *b*-value acquisitions for 3-shell HARDI. Overall, we observe an increase in the reconstruction error with increasing *b*-value (see Figure 3). Compared to signal interpolation, of course, higher NMSE was expected for signal extrapolation, yet not to this extent, since Ning et al. (2015) report lower NMSE for signal extrapolation using this method. We argue that an NMSE of up to 30% due to signal extrapolation and reduced SNR at higher *b*-values for 3-shell HARDI is not favorable for subsequent diffusion and microstructural parameter estimation. Thus, we did not include results from sparse reconstruction for the 3-shell HARDI scheme in our dMRI analysis. We acknowledge, however, that 3-shell HARDI variants specifically tailored for high *b*-values (e.g., including *b* = 6,800 s/mm^2^) would avoid signal extrapolation and may thus reduce the NMSE at high *b*-values compared to Figure 3.

### 4.2. DMRI analysis

#### 4.2.1. Diffusion and microstructural parameter estimation

Diffusion parameter maps and the quantitative statistical analysis across all subjects show good agreement between CS-DSI and 3-shell HARDI for diffusion models processing DWIs with *b*-values up to approx. 3,000 s/mm^2^. Overall, diffusion parameters stemming from distinct WM tracts can be well distinguished for both CS-DSI and 3-shell HARDI and are not masked by differences in the dMRI protocols. For the DT model, a slight upward FA bias of CS-DSI compared to 3-shell HARDI is explained by reduced SNR due to longer TE for higher *b*-value acquisitions (Farrell et al., 2007). Slightly lower residual errors from the tensor and NODDI model fitting for 3-shell HARDI compared to CS-DSI support this finding. Additionally, the 3-shell HARDI protocol allows us to fit the DT model to the 30 DWIs of the inner shell at *b* = 1,000 s/mm^2^ (Table 1). In contrast, a subset of 28 CS-DSI DWIs is used to fit the DT model to measurements with multiple *b*-values in the range of 230–1,300 s/mm^2^ (Table 1). This may lead to a different fit to the mono-exponential signal decay. The resulting difference between CS-DSI and 3-shell HARDI in FA_D_ is confirmed by statistical analysis as depicted in Figure 5 and Table 2. For ODI estimated for both acquisition schemes in the CC WM regions, our results show an anterior-posterior gradient indicated by a decrease in ODI from the genu over the body to the splenium of the CC (Figures 5, 6). In these WM ROIs, an overall increase in ODI is observed for CS-DSI compared to 3-shell HARDI which is supported by the statistical analysis using the linear fixed effects model. This difference might stem from differences in the acquisition scheme, but further investigations are needed. To investigate the significant differences observed for AD_D_ and ODI with fixed effects models, we performed further statistical analyses. As depicted in Figure 5, the biggest differences in AD_D_ and ODI between acquisition schemes occur in the WM regions of the CST and CC, respectively. Running a linear fixed effects model with CST and CC regions excluded for AD_D_ and ODI, respectively, we observe *p*-values above the significance level. This finding confirms that mainly these WM regions affect the overall significance obtained for AD_D_ and ODI by the fixed effects model across all WM ROIs. Fitting the kurtosis model results in more similar DT derived parameters of both protocols. This can be explained by the reduced *b*-value dependency of the kurtosis model compared to the DT model (Veraart et al., 2011). The kurtosis model accounts for the diffusional non-Gaussianity and incorporates the non-monoexponential behavior of the multiple *b*-value CS-DSI acquisitions in the estimation of the diffusion tensor. The similarity between protocols in parameters derived from the kurtosis model is supported by *p*-values above the significance levels across all WM ROIs. An in-house implementation of the kurtosis model that integrates important constraints for robustness to noise (Groeschel et al., 2016) was found to improve the fit and the derived parameter maps. Since DT derived metrics from the DT model and the kurtosis model, however, are not directly comparable (Lanzafame et al., 2016), those parameters should be reported for both models. To further extend the comparison of DT model parameters obtained from CS-DSI and 3-shell HARDI, future work could convert CS-DSI and 3-shell HARDI data to corresponding single-shell HARDI data (Yeh and Verstynen, 2016). In this way, FA and diffusivities can be obtained for CS-DSI and 3-shell HARDI without fitting the tensor model to measurements with multiple *b*-values. Despite the slight SNR reduction and the effects of multi *b*-value data on the DT model fitting, the CS-DSI imaging protocol delivers reliable data. Our results on kurtosis metrics agree with recent findings by Sprenger et al. (2016) who investigate acquisition schemes for diffusion kurtosis imaging (DKI) and report that both 3-shell and CS-DSI schemes are suitable choices. The comparable performance of both schemes with respect to kurtosis model fitting indicates a low dependency of kurtosis parameter estimation on the acquisition scheme as similarly suggested by Sprenger et al. (2016) with respect to the bias on DKI metrics. Further, in contrast to the 3-shell HARDI scheme, CS-DSI provides high *b*-value acquisitions increasing its potential for future image analysis. In this work, we selected the CHARMED model as an example of a state-of-the-art diffusion model that requires DWIs at high *b*-values (> 3,000 s/mm^2^). Our results show that compared to the dedicated CHARMED reference, CS-DSI allows for accurate FR parameter estimation that the standard 3-shell HARDI scheme applied in population imaging cannot provide. Together with the diffusion parameter maps derived from the diffusion propagator, this experiment highlights the advantages of the higher *b*-value CS-DSI acquisitions. Future developments of novel mathematical or biophysical diffusion models might have similar requirements which would be fulfilled by dMRI data collected with the CS-DSI imaging protocol. In the context of a long-term population study, this benefit gained from including scans with higher diffusion weighting in the imaging protocol makes up for the compromise of a slightly reduced SNR (~4% in brain tissue) that, in any case, with the emergence of novel denoising techniques (Veraart et al., 2016b) could potentially be overcome.

Measures for test-retest reliability and repeatability, the ICC and the wsCV, were obtained on a subset of 5 subjects for each of the diffusion protocols of interest. Due to the wide range of questions we aimed to answer, rescans could be collected for just a small sample size. Differences between the subjects, e.g., through subject motion during acquisition, will not be smoothed out across just 5 subjects and will, therefore, influence the TRT evaluation and comparison between CS-DSI and 3-shell HARDI. Despite this limitation of our pilot study data, we report ICC and wsCV values above 0.85 and smaller than 4%, respectively, for both imaging protocols. TRT indices of this scale are in agreement with related work on TRT analysis of diffusion models (Vollmar et al., 2010; Willats et al., 2014) and lie within acceptable ranges for reliability and repeatability. Both CS-DSI and 3-shell HARDI provide similar ICC confirming the validity of both diffusion protocols. Our results, further, show that for both protocols, repeatability decreases with the use of more advanced methods. This may simply be explained by the models' requirements of higher *b*-value acquisitions with lower SNR and increased eddy-current-induced distortions contributing to more differences between scan and rescan. The same effects occurs for high *b*-value CS-DSI data that provides adequate repeatability indices but higher within-subject variability than 3-shell HARDI. Our results on test-retest repeatability point toward a similar direction as the findings by Sprenger et al. (2016) on the superior performance of multi-shell schemes in terms of bias and precision. However, due to the limitations of our pilot study, which influence TRT analysis, further investigation in this regard is necessary. Additionally, for CS-DSI, the TRT results show improved repeatability of diffusivities when using the kurtosis rather than the DT model. This supports our earlier findings from the group averaged diffusion parameters indicating that, for multiple *b*-value CS-DSI acquisitions, the kurtosis model is better suited for the estimation of DT derived parameters than the tensor model.

#### 4.2.2. Fiber orientation and macroscopic brain connections

Three state-of-the-art methods for the estimation of fiber orientations which form the basis for tractography were evaluated and compared for both CS-DSI and 3-shell HARDI. Visual comparison suggests that comparable orientational information is provided by CS-DSI and 3-shell HARDI. Specifically, for 3-shell HARDI, fODFs estimated via (Ankele et al., 2017) are in good agreement with fODFs obtained by means of the state-of-the-art CSD approach. Thus, we based the quantitative comparison of the orientational information obtained for CS-DSI and 3-shell HARDI on the fODFs estimated using the former method. In this regard, the similarity of the orientational information obtained from both advanced dMRI acquisitions and their robustness across subjects is quantified by a high angular cross-correlation with low standard error. Additionally, the high angular cross-correlation coincides with the small angular deviation in the estimated fiber directions and in the crossing angle in multi-fiber voxels. An adequate minimum crossing angle can be resolved by each of the advanced acquisition schemes. The reported percentages of 1/2/3 fiber voxels within the applied WM mask agree with previous works (Schultz, 2012; Jeurissen et al., 2013). We do not compute this metric per acquisitions scheme due to the lack of a protocol-specific criterium for choosing the threshold used for fiber selection. Due to this limitation, Figure 10 shows small variations in the estimated number of fibers between CS-DSI and 3-shell, which are, however, on average below 1 fiber in WM. Additionally, comparable tissue volume fraction maps for WM, GM and CSF are obtained for CS-DSI and 3-shell HARDI, which can provide guidance for fiber tracking equally well for both acquisition schemes. Thus, despite acquiring dMRI data with different diffusion protocols and employing different methods for ODF reconstruction, comparable fiber orientations of the brain microstructure can be estimated from both CS-DSI and 3-shell HARDI. Supporting these results, probabilistic fiber tracking based on the standard FSL pipeline further indicates good agreement of fiber orientation estimates from both dMRI protocols. Recent investigations by Gigandet et al. (2013) on the dependency of brain network connectivity measures on different acquisitions schemes agree with our finding that DSI acquisitions are at least as suitable as 3-shell HARDI scans for the extraction of orientational information, although both schemes are conceptually very different. Gigandet et al. (2013) further report the highest connectivity for a DSI acquisitions scheme similar to the one applied in this work.

### 4.3. CS-DSI in population imaging

This work primarily focuses on showing that for a wide range of state-of-the-art diffusion analysis techniques, CS-DSI is able to provide diffusion information similar to that captured by 3-shell HARDI, despite the small SNR loss due to higher *b*-value acquisitions. This is the starting point for validating the applicability of CS-DSI for population imaging. Note that in this work we do not aim to decide whether CS-DSI or a multi-shell scheme is more suitable for population imaging. We rather chose the latter as a gold standard protocol. The CS-DSI protocol proposed in this work for population imaging should at least deliver dMRI data comparable to that obtained by conventional and well-established dMRI protocols such as 3-shell HARDI. Our pilot study affirms the above for CS-DSI and 3-shell HARDI acquisitions collected at both 1.5 and 2.0 mm isotropic resolution. Here, we only present the results for 1.5 mm isotropic resolution as we observed the same trends for both resolutions. Furthermore, the collection of CS-DSI acquisitions allows for high flexibility in dMRI analysis as already noted in previous works such as Sprenger et al. (2016). First, CS-DSI contains high *b*-value acquisitions and thus provides dMRI data applicable to diffusion models that specifically require high diffusion weightings and with a great potential to be well-suited for future methods developed for dMRI analysis (Veraart et al., 2016a). Second, complimentary biomarkers can be extracted directly from the diffusion propagator which is obtained in a model-free manner by CS-DSI without any prior assumptions. Diffusion measures such as the return-to-origin probability and the return-to-axis probability as well as the propagator anisotropy and non-Gaussianity have already been proposed to map the microstructure of brain tissue (Özarslan et al., 2013). Also, the clinical feasibility of estimating those parameters from subsampled MR data has recently been reported (Avram et al., 2016). Future developments might further exploit the diffusion information content provided by the diffusion propagator. In this work, we additionally determined propagator-based diffusion parameters and CHARMED parameters to highlight and quantify the potentials of CS-DSI to provide dMRI data well-suited for such analysis which further extends this protocol's scope of application for population imaging.

## 5. Conclusion

The contributions of this work are three-fold: (1) defining an imaging protocol for compressed sensing DSI complying with stringent scan time limits of long-term population studies, (2) conducting a pilot study delivering dMRI data for an in depth investigation of this protocol based on a wide spectrum of state-of-the-art diffusion processing and analysis techniques, and (3) validating the applicability and potentials of accelerated DSI using CS for population imaging. The results of this work indicate comparable performance of CS-DSI and the 3-shell HARDI scheme well-established in population imaging in the estimation and reliability of diffusion and microstructural parameters when considering DWIs up to the same maximum diffusion weighting for both protocols. Further, both schemes perform comparable in the inference of fiber orientation and macroscopic brain connections. These findings hold despite the slightly smaller SNR of CS-DSI scans due to higher *b*-value acquisitions. Providing such high *b*-value data, CS-DSI enables the accurate fitting of specific microstructure models such as the CHARMED model. Additionally, the diffusion propagator obtained by means of the model-free DSI approach allows for high quality fiber orientation estimation and the extraction of further complimentary biomarkers. Even if stringent scan time limits are imposed, CS-DSI provides high radial resolution while maintaining high angular resolution and it is a forward-looking acquisition strategy with a great potential for future developments. Thus, CS-DSI presents a well-suited imaging protocol for dMRI within the scope of a scan time-limited, high throughput, long-term population study.

## Author contributions

AT, RS, ThS, AR, MB, and ToS designed research. AT, RS, RH, ThS, and AR contributed sequences and analysis tools. AT and RS collected and analyzed data. AT, RS, ThS, AR, MB, and ToS interpreted data. AT wrote manuscript. AT, RS, RH, ThS, AR, MB, and ToS reviewed and approved manuscript.

### Conflict of interest statement

The authors declare that the research was conducted in the absence of any commercial or financial relationships that could be construed as a potential conflict of interest.
